# Neuroprotective Effect of a New Synthetic Aspirin-decursinol Adduct in Experimental Animal Models of Ischemic Stroke

**DOI:** 10.1371/journal.pone.0074886

**Published:** 2013-09-20

**Authors:** Bing Chun Yan, Joon Ha Park, Bich Na Shin, Ji Hyeon Ahn, In Hye Kim, Jae-Chul Lee, Ki-Yeon Yoo, In Koo Hwang, Jung Hoon Choi, Jeong Ho Park, Yun Lyul Lee, Hong-Won Suh, Jong-Gab Jun, Young-Guen Kwon, Young-Myeong Kim, Seung-Hae Kwon, Song Her, Jin Su Kim, Byung-Hwa Hyun, Chul-Kyu Kim, Jun Hwi Cho, Choong Hyun Lee, Moo-Ho Won

**Affiliations:** 1 Institute of Integrative traditional & western Medicine，Medical College, Yangzhou University, Yangzhou, China; 2 Department of Neurobiology, School of Medicine, Kangwon National University, Chuncheon, South Korea; 3 Department of Physiology, College of Medicine and Institute of Neurodegeneration and Neuroregeneration, Hallym University, Chuncheon, South Korea; 4 Department of Oral Anatomy, College of Dentistry, Gangneung-Wonju National University, Gangneung, South Korea; 5 Department of Anatomy and Cell Biology, College of Veterinary Medicine and Research Institute for Veterinary Science, Seoul National University, Seoul, South Korea; 6 Department of Anatomy, College of Veterinary Medicine, Kangwon National University, Chuncheon, South Korea; 7 Division of Applied Chemistry and Biotechnology, Hanbat National University, Daejeon, South Korea; 8 Department of Pharmacology and Institute of Natural Medicine, College of Medicine Hallym University, Chuncheon, South Korea; 9 Department of Chemistry and Institute of Applied Chemistry, Hallym University, Chuncheon, South Korea; 10 Department of Biochemistry, College of Life Science and Biotechnology, Yonsei University, Seoul, South Korea; 11 Vascular System Research Center and Department of Molecular and Cellular Biochemistry, School of Medicine, Kangwon National University, Chuncheon, South Korea; 12 Division of Analytical Bio-imaging, Chuncheon Center, Korea Basic Science Institute, Chuncheon, Kangwon, South Korea; 13 Molecular Imaging Research Center, Korea Institute of Radiological and Medical Sciences, Seoul, South Korea; 14 Laboratory Animal Center, OSONG Medical Innovation Foundation, Osong, South Korea; 15 Department of Emergency Medicine and Institute of Medical Sciences, Kangwon National University Hospital, School of Medicine, Kangwon National University, Chuncheon, South Korea; 16 Department of Anatomy and Physiology, College of Pharmacy, Dankook University, Cheonan, South Korea; University of Münster, Germany

## Abstract

Stroke is the second leading cause of death. Experimental animal models of cerebral ischemia are widely used for researching mechanisms of ischemic damage and developing new drugs for the prevention and treatment of stroke. The present study aimed to comparatively investigate neuroprotective effects of aspirin (ASA), decursinol (DA) and new synthetic aspirin-decursinol adduct (ASA-DA) against transient focal and global cerebral ischemic damage. We found that treatment with 20 mg/kg, not 10 mg/kg, ASA-DA protected against ischemia-induced neuronal death after transient focal and global ischemic damage, and its neuroprotective effect was much better than that of ASA or DA alone. In addition, 20 mg/kg ASA-DA treatment reduced the ischemia-induced gliosis and maintained antioxidants levels in the corresponding injury regions. In brief, ASA-DA, a new synthetic drug, dramatically protected neurons from ischemic damage, and neuroprotective effects of ASA-DA may be closely related to the attenuation of ischemia-induced gliosis and maintenance of antioxidants.

## Introduction

Stroke is developed due to ischemia and hemorrhage in the brain and it is the second leading global cause of death [1,2]. About 87% of patients with stroke are caused by ischemia, and ischemic stroke is induced by a loss of blood supply to part of the brain [3]. Prevention of ischemic stroke is thus of considerable importance [4,5,6,7], and it should be solved by some antiplatelet drugs, such as aspirin, which are commonly used in daily clinical settings for preventing stroke, recently [8].

Acetylsalicylic acid (Aspirin, ASA), a nonsteroidal anti-inflammatory drug, has various pharmacological activities, and has been widely used for prevention and treatment of stroke. ASA is known to be useful in the management of patients with cerebral ischemia, due to its anti-thrombotic as well as direct neuroprotective effect [9]. In addition, many studies have showed neuroprotective effects of ASA, including derivatives of ASA, in various ischemic models [9,10].

Angelica gigas Nakai, which has been used in oriental traditional medicine, exerts some pharmacological effects including the inhibition of acetylcholinesterase [11]. Decursinol (DA) is known as one of the coumarins purified from dried roots of *Angelica* *gigas* Nakai, and has diverse pharmacological activities such as anti-nociceptive activity, anti-amnesic activity and anti-amyloid β protein aggregation [12,13,14]. In addition, DA has neuroprotective activity against glutamate-induced neurotoxicity [15].

Experimentally induced transient cerebral ischemia including global and focal cerebral ischemia, can be induced by the occlusion of related artery or arteries, and leads to extensive neuronal degeneration and/or death in some brain regions [16]. Transient global cerebral ischemia easily causes neuronal death/damage in the hippocampal CA1 region, which is known to be the most vulnerable region to transient cerebral ischemic damage, and selective neuronal death in the CA1 region occurs some days after initial ischemic insult, which is referred to as “delayed neuronal death” [17]. Transient focal ischemia induced by middle cerebral artery occlusion (MCAO) produces an infarct of varying size, and has been well used for a neurological and pathological evaluation of a reproducible model [18]. Both animal models have been widely used for researching the molecular mechanism of ischemic damage and developing new drugs for prevention and treatment of stroke [19,20].

Many investigators have focused on protective effects of ASA and DA against neuronal damage induced by various insults including cerebral ischemia. However, few comparative studies regarding neuroprotective effects of ASA and DA against transient global and focal cerebral ischemia have been reported. Especially, no studies have been focused on neuroprotective effects of new synthesized aspirin-decursinol adduct (ASA-DA) against transient global and focal cerebral ischemic damage. In the present study, therefore, we comparatively examined the neuroprotective effects of ASA, DA and ASA-DA against experimentally induced transient focal and global cerebral ischemic damage.

## Materials and Methods

### Synthesis of ASA-DA

**Figure 1:**
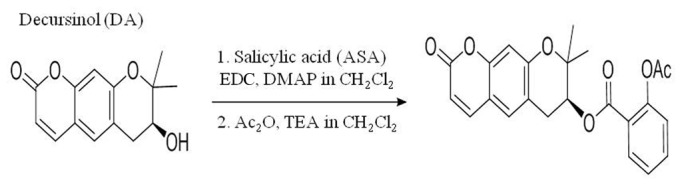
Synthesis of ASA-DA.

The synthesis of ASA-DA (S)-2,2-dimethyl-8-oxo-2,3,4,8-tetrahydropyrano[3,2-g] chromen-3-yl 2-acetoxybenzoate was carried out as follows (Figure 1). A mixture of salicylic acid (0.67 g, 4.9 mmol), EDC (1.0 g, 5.4 mmol) and DMAP (0.50g, 1.2 mmol) dissolved in CH2Cl2 (30 mL) was stirred for 30 min at rt under Ar atmosphere. (+)-Decursinol (0.60 g, 2.4 mmol) solution in CH2Cl2 (3 mL) was added to the activated salicylic acid solution. The reaction mixture was refluxed for 24 h and then cooled down to rt. The combined organic extract was dried over anhydrous MgSO4. The organic solvent was removed under vacuum and the decursinol-salicylic acid ester (640 mg, 72% yield) was isolated from column chromatography (ethyl acetate: hexane =1:3). The purified ester was directly applied for acetylation reaction. To the mixture of decursinol-salicylic acid ester (500 mg, 1.37 mmol), DMAP (33 mg, 0.27 mmol), and TEA (0.95 mL, 6.8 mmol) in CH2Cl2 (15 mL) acetic anhydride (0.52 mL, 5.5 mmol) was added at rt under Ar atmosphere. The mixture was stirred for 4 h at rt. Ethanol (10 mL) and water (20 mL) were added to the solution. The aqueous layer was extracted with CH2Cl2 (20 mL x 2). The combined organic layer was dried over anhydrous MgSO4. The organic solvent was removed under vacuum and the decursinol-aspirin (400 mg, 72% yield) was isolated from column chromatography (ethyl acetate: hexane=1:2) (Figure 1). m.p. 82-83°C, 1H NMR (400 MHz, CDCl3) δ 1.41 (s, 3H), 1.47 (s, 3H), 2.31 (s, 3H), 2.96 (dd, J=4.4, 17.2 Hz, 1H), 3.27 (dd, J =4.4, 17.2 Hz, 1H), 5.27 (t, J =4.4 Hz, 1H), 6.24 (d, J =9.6 Hz, 1H), 6.84 (s, 1H), 7.09 (d, J =8.0 Hz, 1H), 7.17 (s, 1H), 7.29 (d, J =8.0Hz, 1H), 7.56 (dt, J =1.6, 8.0 Hz, 1H), 7.58 (d, J =9.6 Hz, 1H), 7.91 (dd, J =1.6, 8.0 Hz, 1H).

### Experimental animals

Male Sprague-Dawley rats (8 weeks, B.W., 260-270 g) were purchased from Charles River Laboratories (Seoul, Korea). Male Mongolian gerbils (*Meriones unguiculatus*) were obtained from the Experimental Animal Center, Hallym University, Chuncheon, South Korea. Gerbils were used at 6 months (B.W., 65-75 g) of age. The animals were housed in a conventional state under adequate temperature (23°C) and humidity (60%) control with a 12-h light/12-h dark cycle, and provided with free access to water and food. Animals were acclimated to their environment for 5 days before use for experiments. All experimental procedures were in accordance with the National Institutes of Health guidelines and ARRIVE guidelines for the care and use of laboratory animals. The animal protocol used in present study was reviewed and approved based on ethical procedures and scientific care by the Kangwon National University-Institutional Animal Care and Use Committee (KIACUC-12-0010).

### Treatment with ASA, DA and ASA-DA

To elucidate the neuroprotective effects of ASA, DA and ASA-DA against ischemic damage, the experimental design the experimental animals (rats for focal ischemia and gerbils for global ischemia) were randomly divided into 15 groups as follows (n = 7 in each group); 1) vehicle (dimethyl sulfoxide, DMSO)-treated sham-operated group (vehicle-sham-group), 2) vehicle-pre- and post-treated ischemia-operated groups (vehicle-pre- and post-ischemia-group), 3) 10 and 20 mg/kg ASA-pre- and post-treated ischemia-operated groups (ASA-pre- and post-ischemia-group), 4) 10 and 20 mg/kg DA-pre- and post-treated ischemia-operated groups (DA-pre- and post-ischemia-group), 5) 10 and 20 mg/kg ASA-DA-pre- and post-treated ischemia-operated groups (ASA-DA-pre- and post-ischemia-group). ASA, DA or ASA-DA were dissolved in DMSO and diluted to the desired concentration with saline. Vehicle, ASA, DA or ASA-DA were administered intraperitoneally once daily for 3 days before ischemic surgery. And post-treated experimental groups were administrated 30 min, 12 and 24 h after the ischemic surgery.

To further elucidate the mechanism of neuroprotective effect of ASA-DA against ischemic damage, the gerbils were divided into 6 groups as following (n = 7 in each group): 1) vehicle-sham-group, 2) vehicle-ischemia-group at 2 days post-ischemia, 3) vehicle-ischemia-group at 5 days post-ischemia, 4) 20 mg/kg ASA-DA-sham-group, 5) 20 mg/kg ASA-DA-post-ischemia-group at 2 days post-ischemia, 6) 20 mg/kg ASA-DA-post-ischemia-group at 5 days post-ischemia. ASA-DA was dissolved by same method with above. In addition, with our experimental design, the preliminary test was carried out for the calculation of the sample size and statistical power in order to reduce the bias in the design.

### Transient focal cerebral ischemia

Introduction of transient focal cerebral ischemia: Rats were initially anesthetized with 3.0% isoflurane in a 70% N2O and 30% O2 (v/v) mixture via face mask. Anesthesia was maintained with 2% isoflurane. A rectal temperature probe was introduced, and a heating pad maintained the body temperature at 37°C during whole surgery period. Focal cerebral ischemia was achieved by middle cerebral artery occlusion (MCAO) on the right-side (Belayev et al., 1996). Briefly, the right carotid artery was exposed through a midline cervical incision. The right external carotid artery was dissected free and isolated distally by coagulating its branches and placing a distal ligation prior to transection. A piece of 3-0-monofilament nylon suture (Ethicon, Johnson-Johnson, Brussels, Belgium), with its tip rounded by gentle heating and coated by 0.1% (w/v) poly-L-lysine, was inserted into the lumen of right ECA stump and gently advanced 17.5 mm into the internal carotid artery (ICA) from the bifurcation to occlude the ostium of MCA. After 2 h of ischemia, the suture was pulled back and the animals were allowed to recover. Sham-operated animals were subjected to the same surgical procedures except that the ICA were not inserted

Measurement of PET-CT and Rotarod test: Positron-emission tomography (PET) evaluation of brain function was carried out and cerebral glucose metabolism was measured to evaluate brain function on day one after MCAO. In detail, The animals were injected with 100 µCi of 2-deoxy-2-[18F] fluoro-D-glucose (FDG) through the tail vein and imaged using a small-animal PET scanner (Inveon PET; Siemens). Images were acquired for 10 min under inhalation anesthesia (isoflurane, 2%). FDG/PET-CT images were reviewed using fusion software (Inveon, Siemens, Knoxville, TN). PET-CT and fused whole-body images were displayed in axial, coronal, and sagittal planes, and they were available for review. The level of radioactivity in brain tissue (percentage dose per gram) was estimated from the images according to the method published by Hsieh et al. [21].

A rotating rod apparatus (M.T6800, Borj Sanat, Iran) was used to assess motor performance and measure the ability of rats to improve motor skills with training. The rats were placed on an elevated (height 30 cm) accelerating rod beginning at 5 rpm/min for three consecutive times. Each session lasted a maximum of 200 s, during which time the rotating rod underwent a linear acceleration from 5 to 50 rpm over the first 120 s of the trial and then remained at the maximum speed for the remainder of 200 s. The animals were scored for their latency (in seconds) to fall for each trial. The rats were given a minimum rest of 30 min between trials to avoid fatigue.

Measurement of infarct volume: After measurements of cerebral glucose metabolism, the animals were anesthetized with chloral hydrate and decapitated. Their brains were cut into coronal slices of 2 mm in thickness using a rat brain matrix (Ted Pella, Redding, CA, USA). The brain slices were then incubated in 2% 2,3,5-triphenyltetrazoliumchloride (TTC, Sigma, St. Louis, MO, USA) at 37 °C for 30 min to reveal the ischemic infarction. After TTC reaction, the brain slices were fixed with 4% paraformaldehyde (pH 7.4) in 0.1 M phosphate buffer (PB) for one day and subsequently cryoprotected in PB containing 30% sucrose at 4 °C for two days. The cross-sectional area of infarction between the bregma levels of +4 mm (anterior) and -6 mm (posterior) were determined with a computer-assisted image analysis program (OPTIMAS 5.1, BioScan Ins., USA). On each slice, the brain infarct size was measured manually by outlining the margins of infarct areas, and the infarct volume was then calculated according to the slice thickness of 2 mm per section. Each side of the brain slices was measured separately, and mean values were calculated. The total volume of infarction was determined by integrating six chosen sections and expressed as percentage of the total brain volume.

### Transient global cerebral ischemia

Induction of transient global cerebral ischemia: Gerbils were anesthetized with a mixture of 2.5% isoflurane (Baxtor, Deerfield, IL) in 33% oxygen and 67% nitrous oxide. Bilateral common carotid arteries were isolated and occluded using non-traumatic aneurysm clips. The complete interruption of blood flow was confirmed by observing the central artery in retinae using an ophthalmoscope. After 5 min of occlusion, the aneurysm clips were removed from the common carotid arteries. The body (rectal) temperature under free-regulating or normothermic (37 ± 0.5°C) conditions was monitored with a rectal temperature probe (TR-100; Fine Science Tools, Foster City, CA) and maintained using a thermometric blanket before, during and after the surgery until the animals completely recovered from anesthesia. Thereafter, animals were kept on the thermal incubator (Mirae Medical Industry, Seoul, South Korea) to maintain the body temperature of animals until the animals were euthanized. Sham-operated animals were subjected to the same surgical procedures except that the common carotid arteries were not occluded.

Spontaneous motor activity: For spontaneous motor activity, gerbils were individually placed in a Plexiglas cage (25 cm × 20 cm × 12 cm), located inside a soundproof chamber. Locomotor activity was also recorded with Photobeam Activity System -Home Cage (San Diego Instruments). The cage was fitted with two parallel horizontal infrared beams 2 cm off the floor. Movement was detected by the interruption of an array of 32 infrared beams produced by photocells. Spontaneous motor activity was monitored during 24 h and, simultaneously, the number of times each animal reared and the time (in seconds) spent in grooming behavior were recorded. Locomotor activity data were acquired by an AMB analyser (IPC Instruments, Berks, U.K.). Results were evaluated in terms of entire distance (meters) traveled for 60 min test period. Scores were generated from live observations, and video sequences were used for subsequent re-analysis.

Tissue processing for histology: For the histological analysis, animals were anesthetized with sodium pentobarbital and perfused transcardially with 0.1 M phosphate-buffered saline (PBS, pH 7.4) followed by 4% paraformaldehyde in 0.1 M phosphate-buffer (PB, pH 7.4). The brains were removed and postfixed in the same fixative for 6 h. The brain tissues were cryoprotected by infiltration with 30% sucrose overnight. Thereafter, frozen tissues were serially sectioned on a cryostat (Leica, Wetzlar, Germany) into 30-µm coronal sections, and they were then collected into six-well plates containing PBS.

Cresyl violet staining: To investigate the morphological changes and neuronal changes, cresyl violet (CV) staining was performed. In brief, the sections were mounted on gelatin-coated microscopy slides. CVacetate (Sigma, MO) was dissolved at 1.0% (w/v) in distilled water, and glacial acetic acid was added to this solution. The sections were stained and dehydrated by immersing in serial ethanol baths, and they were then mounted with Canada balsam (Kanto, Tokyo, Japan).

Fluoro-Jade B (F-J B) histofluorescence: F-J B (a useful marker for neuronal degeneration) histofluorescence staining were carried out according to the method of the previous study [20]. The sections were first immersed in a solution containing 1% sodium hydroxide in 80% alcohol, and followed in 70% alcohol. They were then transferred to a solution of 0.06% potassium permanganate, and transferred to a 0.0004% F-J B (Histochem, Jefferson, AR) staining solution. After washing, the sections were placed on a slide warmer (approximately 50°C), and then examined using an epifluorescent microscope (Carl Zeiss, Göttingen, Germany) with blue (450-490 nm) excitation light and a barrier filter.

Immunohistochemistry: Neuronal nuclei (NeuN), glial fibrillary acidic protein (GFAP), ionized calcium-binding adapter molecule (Iba-1), superoxide dismutase (SOD) 1, SOD 2, catalase (CAT) and glutathione peroxidase (Gpx) immunohistochemistry were done according to the method of the previous study (Lee et al., 2011; Schmued and Hopkins, 2000). In brief, the sections were incubated with diluted mouse anti-NeuN (1:1000, Chemicon, Temecula, CA), anti-GFAP(1:800, Chemicon, Temecular), rabbit anti-Iba-1 (1:500, Wako), anti-CAT (diluted 1:1,000, LabFrontier), sheep anti-SOD1 (diluted 1:1000, Calbiochem), anti-SOD2 (diluted 1:1000, Calbiochem), and sheep anti-Gpx (diluted 1:1000, Chemicon International) and subsequently exposed to biotinylated horse anti-mouse IgG and streptavidin peroxidase complex (1:200, Vector, Burlingame, CA). And they were visualized by staining with 3,3’-diaminobenzidine (Sigma) in 0.1 M Tris-HCl buffer (pH 7.2). To evaluate the neuroprotective effect of ASA, DA or ASA-DA, NeuN-immunoreactive (+) neurons and F-J B positive (+) cells were counted in a 250 × 250 µm square applied approximately at the center of the CA1 using an image analyzing system (software: Optimas 6.5, CyberMetrics, Scottsdale, AZ). The studied tissue sections were selected with 120-μm intervals, and cell counts were obtained by averaging the counts from each animal.

### Statistical analysis

The data shown here represent the means ± SD. All data have shown normal distribution. Differences of the means among the groups were statistically analyzed by one-way analysis of variance (ANOVA) with a post hoc Bonferroni’s multiple comparison test in order to elucidate ischemia-related differences among experimental groups. Statistical significance was considered at P < 0.05.

## Results

Results from focal transient cerebral ischemia in rats

### Physiological parameters

Physiological parameters, including mean arterial blood pressure, blood gases, blood pH and serum glucose, were not significantly different across all groups at 30 min both before MCAO and after reperfusion (data not show).

### Rotarod analysis

**Figure 2:**
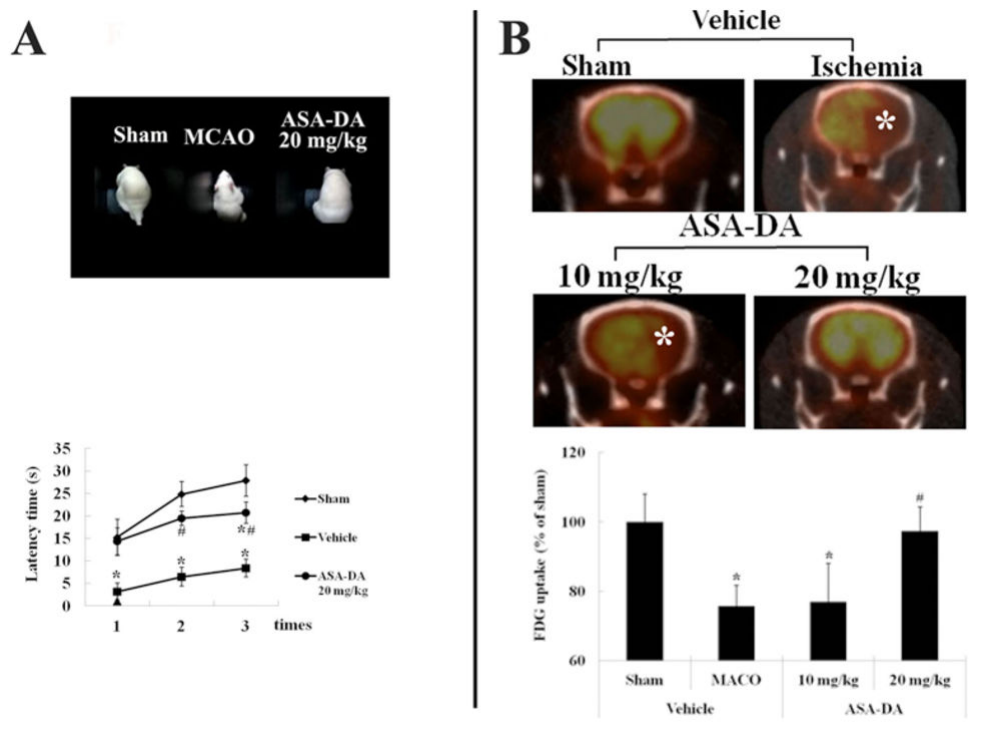
Rotarod test and PET-CT imaging. Rotarod test (A) and PET-CT imaging (B) in the sham-, vehicle-, ASA-DA-pre-ischemia-groups. Only pretreatment with 20 mg/kg ASA-DA significantly ameliorates motor activity and impaired glucose metabolism (asterisks) (n = 7 per group; *P < 0.05, significantly different from the sham-group, #P < 0.05, significantly different from the vehicle-ischemia-group). The bars indicate the means ± SD.

The rotarod test was conducted three consecutive times from one day after MCAO (Figure 2A). A marked difference in latency time was observed between the sham- and ischemia-group. In the 20 mg/kg ADA-DA-ischemia-group, the rats exhibited very strong motor performances that were similar to those in the sham-group.

### PEP-CT analysis

PEP-CT analyses in all the groups are shown in Figure 2B. Brain injury by MCAO dramatically impaired glucose metabolism in the vehicle-ischemia- and 10 mg/kg ASA-DA-ischemia-group. However, treatment with ASA-DA 20 mg/kg significantly ameliorated damage in brain function caused by MCAO.

### TTC staining

**Figure 3:**
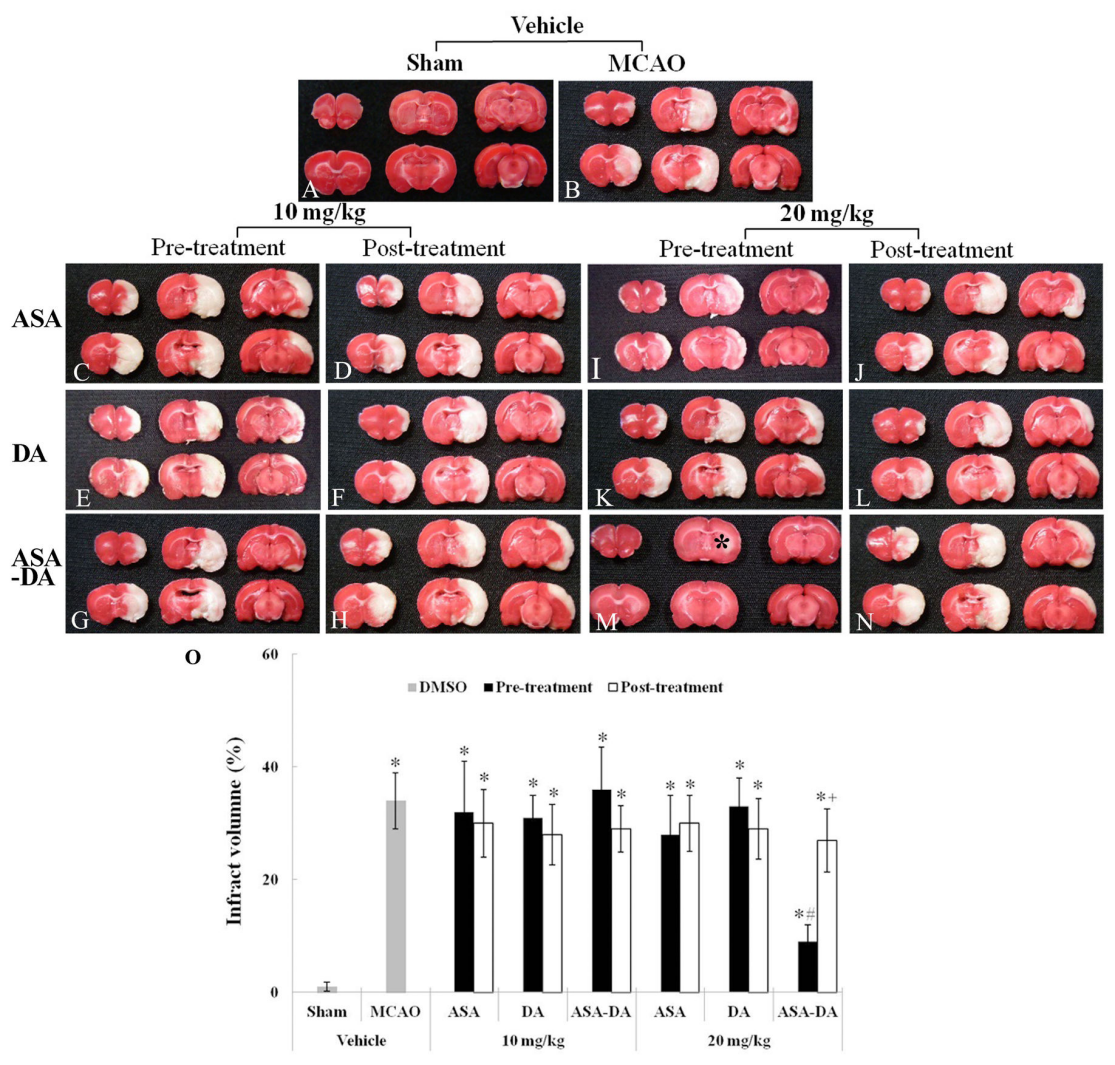
Infarct volume. TTC staining (A-N) in the sham-, vehicle-pre- and post-, 10 and 20 mg/kg ASA, DA and ASA-DA-ischemia-group at 48 h after ischemia-reperfusion. In the vehicle-ischemia-group, infarct areas are apparent. Only in the 20 mg/kg ASA-DA-pre-ischemia-group, the infarct area is notably decreased. Percentage change in infarct volume (O) (n = 7 per group; *P < 0.05, significantly different from the sham-group, #P < 0.05, significantly different from the vehicle-ischemia-group; +P < 0.05, significantly different from the corresponding same treated -group). The bars indicate the means ± SD.

Infarct volume induced by transient focal ischemia was checked by TTC staining (Figure 3). In the vehicle-sham-group, ischemic injury was not shown in any regions (Figure 3A). However, infarct regions were observed in the cerebral cortex and striatum in the vehicle-ischemia-group (Figure 3B). In all the 10 mg/kg ASA, DA and ASA-DA pre- and post-ischemia-groups and in all the 20 mg/kg ASA, DA and ASA-DA post-ischemia-groups, sizes of infarct regions were similar to those of the vehicle-ischemia-group (Figures 3C-H, 3J, 3L, 3N and 3O). In addition, in all the 20 mg/kg ASA, DA and ASA-DA pre-ischemia-groups, we found that the infarct size was significantly decreased only in the ASA-DA pre-ischemia-group (Figures 3I, 3K, 3M and 3O).

Results from global transient cerebral ischemia in gerbils

### SMA analysis

**Figure 4:**
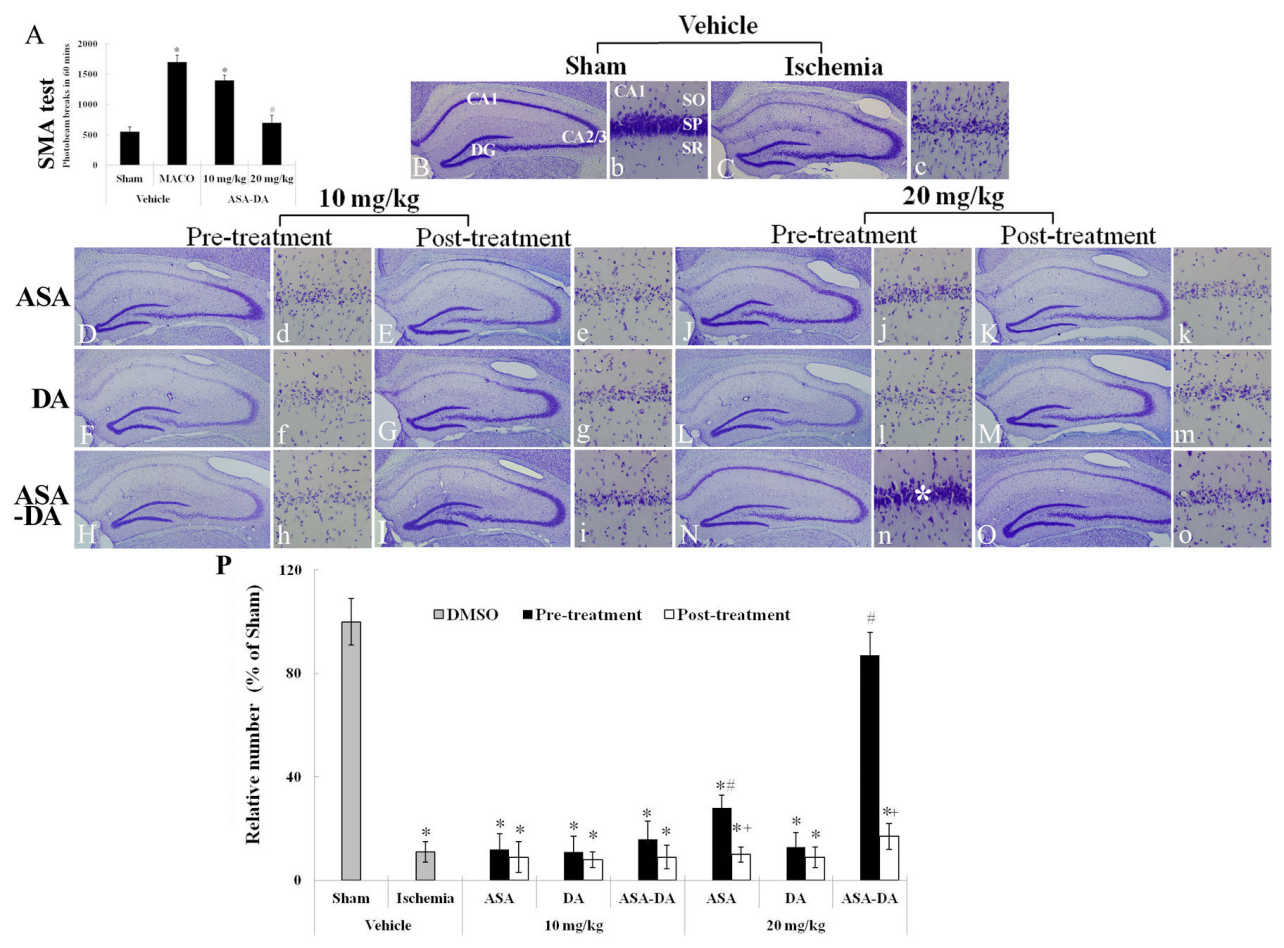
SMA test and CV staining. SMA (A) at 1 day after ischemia-reperfusion in ischemic gerbils and CV staining (B-O) in the gerbil hippocampus at 5 days after ischemia-reperfusion. In the vehicle-ischemia-group, SMA is significantly increased. SMA is much lower only in the 20 mg/kg ASA-DA-ischemia-group. Many CV+ cells were observed only in the 20 mg/kg ASA-DA-pre-ischemia-group (asterisk). CA; conus ammonis, DG; dentate gyrus SO, stratum oriens; SR, stratum radiatum. Scale bar = 50 and 200 µm. Relative analysis (O) as percent in the mean number of CV+ cells in the CA1 region (n = 7 per group; *P < 0.05, significantly different from the vehicle- sham-group, #P < 0.05, significantly different from the vehicle-ischemia-group; +P < 0.05, significantly different from the corresponding same treated -group). The bars indicate the means ± SD.

SMA was examined 1 day after ischemia-reperfusion in ischemic gerbils (Figure 4A). In the vehicle-ischemia-group, SMA was significantly increased compared to that of the vehicle-sham-group. In the 10 mg/kg ASA-DA-ischemia-group, the hyperactivity was similar to that of the vehicle-ischemia-group. However, the activity in the 20 mg/kg ASA-DA-ischemia-group was much lower than that in the vehicle-ischemia-group and similar to that of the vehicle-sham-group.

### CV staining

CV staining was used to observe morphological change in neurons of the stratum pyramidale of the hippocampal CA1 region after transient global ischemia (Figure 4B-4P and 4b-4o)). In the vehicle-sham-group, CV positive (+) cells were easily observed in the stratum pyramidale of the CA1 region (Figure 4B and b). In the vehicle-ischemia-group, a few CV+ cells were found in the stratum pyramidale of the CA1 region (Figures 4C and 4c). In all the 10 mg/kg ASA, DA and ASA-DA pre- and post-ischemia-groups, CV+ cells were not well preserved in the stratum pyramidale of the CA1 region, which was similar to the vehicle-ischemia-group (Figure 4D-4I, 4d-4i and 4P).

Among all the 20 mg/kg pre- and post-ischemia-groups, many CV+ cells were found in the stratum pyramidale of the CA1 region of the 20 mg/kg ASA-DA-pre-treated-ischemia-group (Figures 4N, 4n and 4P). In the other 20 mg/kg pre- and post-ischemia-groups, a few CV+ cells were observed in the stratum pyramidale of the CA1 region (Figures 4J-4M, 4O and 4P).

### NeuN immunohistochemistry and F-J B histofluorescence

**Figure 5:**
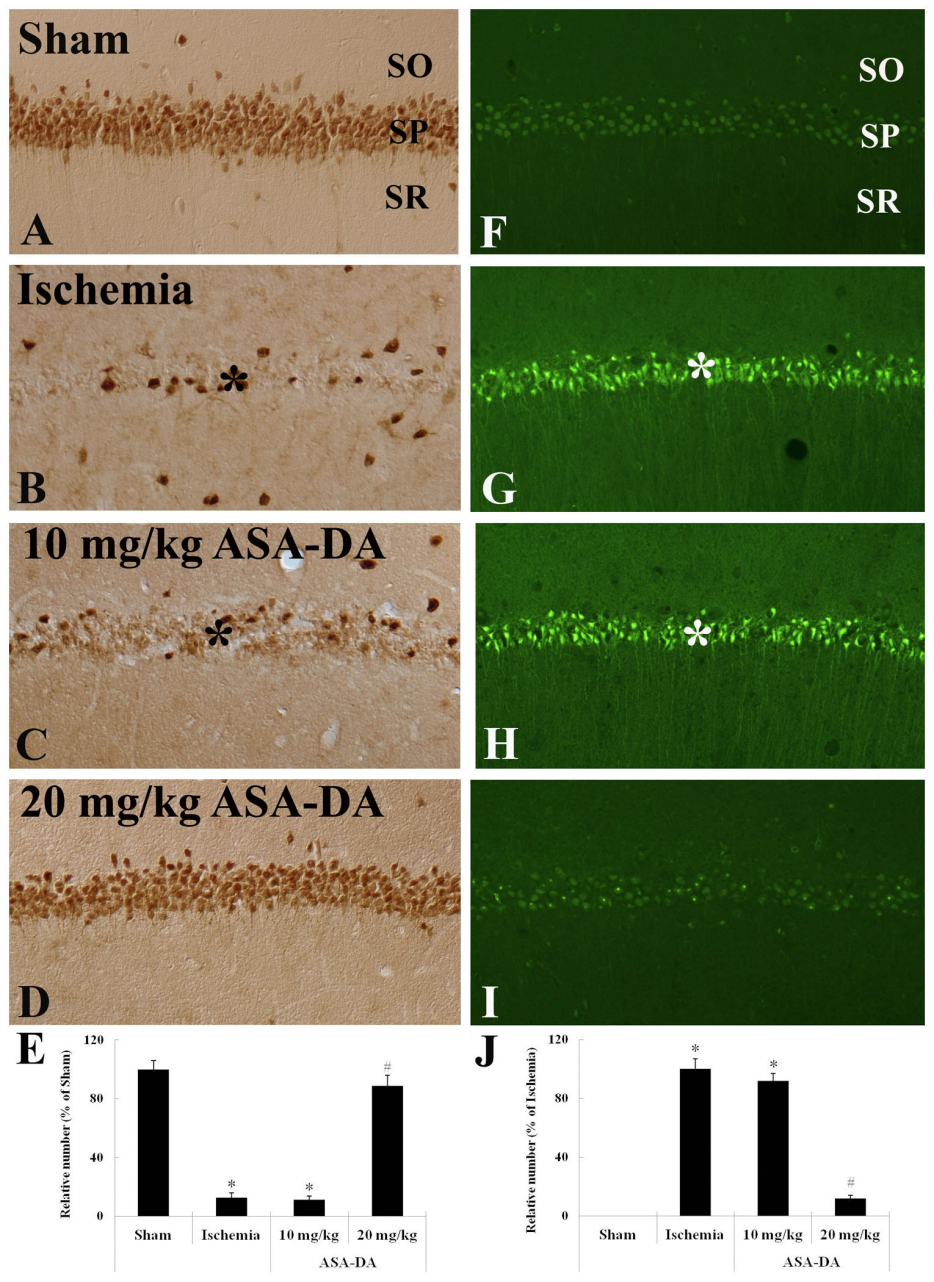
NeuN immunohistochemistry and F-J B histofluorescence. NeuN immunohistochemistry (A-E) and F-J B histofluorescence (F-J) in the CA1 region of the vehicle-sham-, vehicle-ischemia-, 10 and 20 mg/kg ASA-DA-pre-ischemia-groups at 5 days after ischemia-reperfusion. NeuN immunoreactivity is easily detected in the stratum pyramidale (SP) of the CA1 region of the vehicle-sham- and 20 mg/kg ASA-DA-pre-ischemia-groups. In the vehicle-ischemia- and 10 mg/kg ASA-DA-pre-ischemia-groups, many F-J B+ cells are detected in the SP (asterisks). Relative analysis (E and J) as percent in the mean number of NeuN+ and F-J B+ cells in the CA1 region (n = 7 per group; *P < 0.05, significantly different from the vehicle- sham-group, #P < 0.05, significantly different from the vehicle-ischemia-group). The bars indicate the means ± SD.

Neuronal damage/death were shown by NeuN immunohistochemistry and F-J B histofluorescence (Figure 5). In the vehicle-sham-group, abundant NeuN+ neurons were observed in the stratum pyramidale of the CA1 region, and no F-J B+ cells were found in the stratum pyramidale (Figure 5A and 5F). In the vehicle-ischemia-group, most of NeuN+ neurons (about 89% of the sham) disappeared, whereas many F-J B+ cells were detected in the stratum pyramidale (Figures 5B, 5E, 5G and 5J). In the 10 mg/kg ASA-DA-pre-ischemia-group, the pattern of NeuN immunoreactivity and F-J B staining in the stratum pyramidale was similar to that in the vehicle-ischemia-group (Figures 5C, 5E, 5H and 5J). However, NeuN+ neurons and F-J B+ cells in the stratum pyramidale of the 20 mg/kg ASA-DA-pre-ischemia-group were very similar to those of the vehicle-sham-group (Figures 5D, 5E, 5I and 5J).

### Glial activation

**Figure 6:**
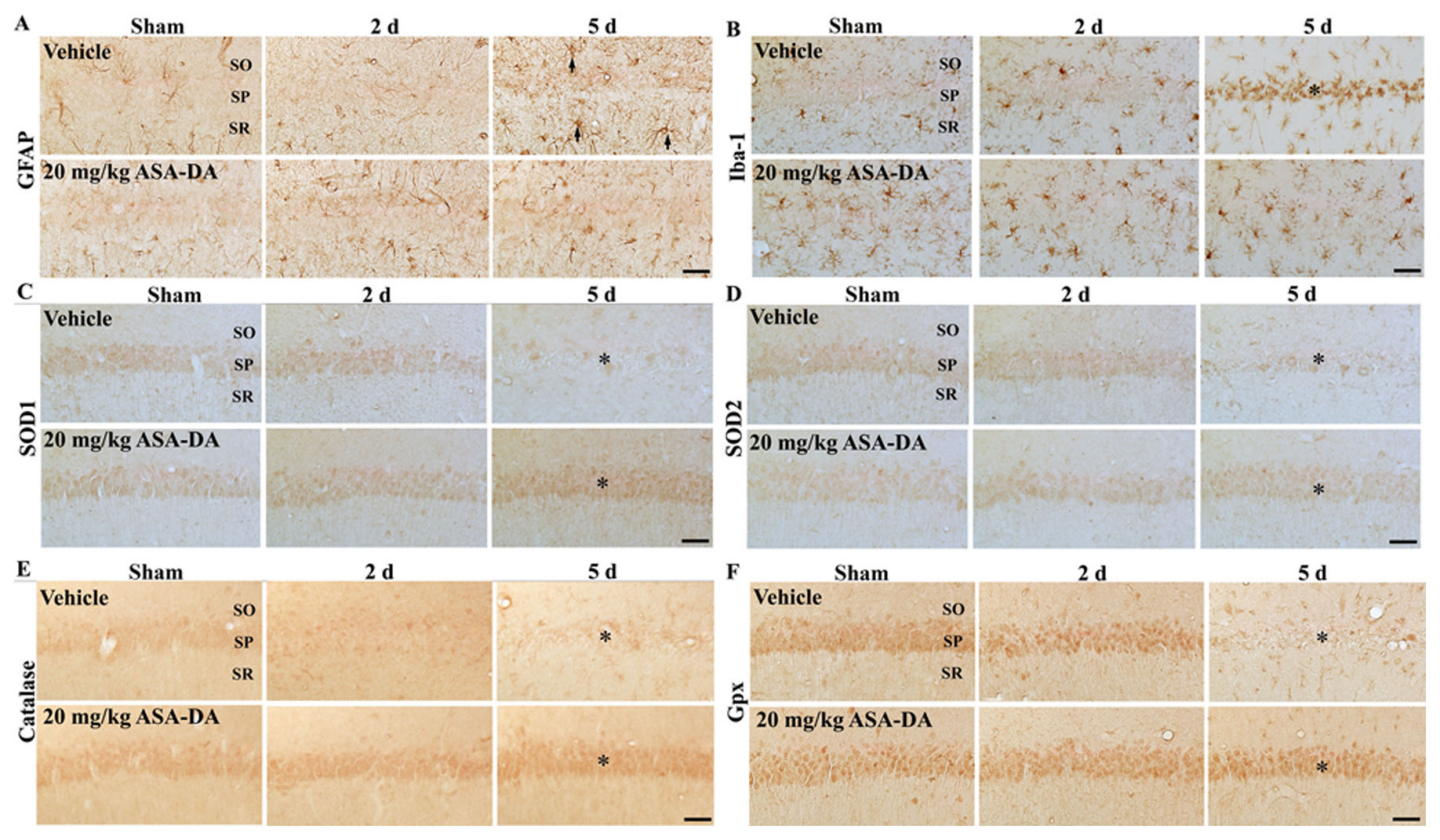
GFAP (A), Iba-1 (B), SOD1 (C), SOD2 (D), CAT (E) and Gpx (F) immunohistochemistry. All of them in the CA1 region of the vehicle- and 20 mg/kg ASA-DA-pre-ischemia-groups at sham, 2 and 5 days after ischemia-reperfusion. In the vehicle-ischemia-group at 5 days post-ischemia, GFAP immunoreactivity increases in all the layers, and Iba-1 immunoreactivity is aggregated in the stratum pyramidale (SP). In the 20 mg/kg ASA-DA-pre-ischemia-group at 5 days post-ischemia, GFAP and Iba-1 immunoreactivity are much lower than those in the vehicle-ischemia-group at 5 days post-ischemia-group. In the vehicle-ischemia-group at 5 days post-ischemia, SOD1, 2, CAT and Gpx immunoreactivity are weakly detected in the stratum pyramidale. However, their immunoreactivity is well maintained or increased in the stratum pyramidale of the 20 mg/kg ASA-DA-ischemia-group at 5 days post-ischemia. SO; stratum oriens, SR; stratum radiatum. Scale bar = 50 µm.

GFAP immunoreactivity (Table 1, Figure 6A): In the vehicle-sham-group, GFAP+ astrocytes showed a resting form and were distributed throughout the CA1 region. In the vehicle-ischemia-group at 2 days post-ischemia, processes of GFAP+ astrocytes became slightly slender. However, GFAP+ astrocytes became reactive form and their immunoreactivity was increased in the vehicle-ischemia-group at 5 days post-ischemia compared with that in the vehicle-sham-group.

**Table 1:** Semi-quantification of immunoreactivities of glial markers and antioxidants in the hippocampal CA1 region of the vehicle-ischemia- and 20 mg/kg ASA-DA-ischemia-group.

Antibodies	Groups	Category	Time after ischemia/reperfusion			
			Sham		2 d	5 d
GFAP	Vehicle	CSP	±		±	+
		CSOR	+		+	++
	20 mg/kg ASA-DA	CSP	±		±	±
		CSOR	±		+	+
Iba-1	Vehicle	CSP	±		±	++
		CSOR	+		++	++
	20 mg/kg ASA-DA	CSP	+		+	+
		CSOR	+		+	+
SOD 1	Vehicle	CSP	+		+	−
		CSOR	±		±	+
	20 mg/kg ASA-DA	CSP	+		+	++
		CSOR	±		±	±
SOD 2	Vehicle	CSP	+		+	−
		CSOR	±		±	+
	20 mg/kg ASA-DA	CSP	+		+	+
		CSOR	±		±	±
CAT	Vehicle	CSP	±		±	−
		CSOR	±		±	+
	20 mg/kg ASA-DA	CSP	+		+	++
		CSOR	±		±	+
Gpx	Vehicle	CSP	++		++	−
		CSOR	±		+	+
	20 mg/kg ASA-DA	CSP	++		++	++
		CSOR	+		±	+

Immunoreactivity is scaled as − ±, + ++ or +++ representing no staining, weakly positive, moderate, strong or very strong, respectively. CSP, cells in stratum pyramidale; CSOR, cells in stratum oriens and *radiatum*.

In the 20 mg/kg ASA-DA-sham-group, GFAP immunoreactivity was a little lower compared with that in the vehicle-sham-group. Also, GFAP immunoreactivity was a little lower in the 20 mg/kg ASA-DA-ischemia-group at 5 days post-ischemia compared with that in the vehicle-ischemia-group.

Iba-1 immunoreactivity (Table 1, Figure 6B): In the vehicle-sham-group, Iba-1+ microglia showed a resting form, and the immunoreactivity was increased at 2 days post-ischemia. At 5 days post-ischemia, strong Iba-1+ microglia were increased and aggregated in the stratum pyramidale of the CA1 region.

In the 20 mg/kg ASA-DA-sham-group, Iba-1 immunoreactivity was a little higher than that in the vehicle-sham-group. In the 20 mg/kg ASA-DA-ischemia-group at 2 and 5 days post-ischemia, Iba-1 immunoreactivity was very similar to that in the 20 mg/kg ASA-DA-sham-group.

### Antioxidants immunoreactivities

SOD1 and 2 immunoreactivities (Table 1, Figures 6C and 6D): Moderate SOD1 and 2 immunoreactivities were found in the stratum pyramidale in the vehicle-sham-group. Their immunoreactivities were apparently decreased at 5 days post-ischemia.

In the 20 mg/kg ASA-DA-sham-group, SOD1 and 2 immunoreactivities in the stratum pyramidale were similar to those in the vehicle-sham-group, and their immunoreactivities at 2 and 5 days post-ischemia were not changed compared with those in the 20 mg/kg ASA-DA-sham-group.

CAT and Gpx immunoreactivities (Table 1, Figures 6E and 6F): In the vehicle-sham-group, weak CAT and moderate Gpx immunoreactivity were observed in the stratum pyramidale of the CA1 region. Their immunoreactivities in the stratum pyramidale were decreased at 2 days post-ischemia, and nearly disappeared at 5 days post-ischemia.

In the 20 mg/kg ASA-DA-sham-group, CAT, not Gpx, immunoreactivity in the stratum pyramidale was a little higher than that in the vehicle-sham-group. At 5 days post-ischemia, CAT, not Gpx, immunoreactivity in the stratum pyramidale was strong compared with that in the 20 mg/kg ASA-DA-sham-group.

## Discussion

Currently, there are available animal models that are geared to stroke researched field. Experimental researches on stroke have contributed invaluable insight into molecular, cellular and systemic pathophysiology of stroke through a variety of different animal models of stroke [2]. In addition, it has been known that drugs, which show substantial efficacy in animal models of cerebral ischemia, could improve outcome in human stroke; rigorous experimental designs and statistical analyses in preclinical experiments are necessary to develop effective therapies in clinical patients [22]. Therefore, in the present study, we used animal models of focal and global cerebral ischemia to more precisely study the neuroprotective effect of new synthetic drug.

Antiplatelet drugs, such as ASA and its derivatives, are commonly used in the clinical setting to prevent stroke. In this study, the neuroprotective effects of ASA, DA and ASA-DA against ischemic damage were examined after transient focal and global cerebral ischemia. Previous studies have shown neuroprotective effects of ASA in models of stroke. For instance, 30 mg/kg, not 10 mg/kg, ASA decreased infarct volume when it was administered immediately after focal cerebral ischemia in rats [23]. Repeated injection with 40 mg/kg, not 20 mg/kg in rats, ASA up to 4 days after MCAO reduced infarct volume significantly, whereas single injection at 30 min after ischemic onset did not [24]. Pre- and post-ischemic treatment with 5 mg/kg ASA for 21 days significantly attenuated spatial memory impairment and neuronal death in the hippocampal CA1 region in rats subjected to repeated transient global cerebral ischemia [25]. Another previous study showed that pre-treatment with 10 mg/kg ASA for 3 days did not have any effect on infarct size and neurological deficits in a mouse model of transient focal cerebral ischemia [26]. In addition, a recent study reported that post-ischemically administered 40 mg/kg ASA led to a significant reduction in infarct volume only in temporary but not in permanent cerebral ischemia [27]. In this study, we also found that 10 and 20 mg/kg ASA could not protect against ischemia-induced neuronal death/damage in models of transient focal and global cerebral ischemia.

On the other hand, it was reported that DA protected primary cultured rat cortical cells from kainic acid- and N-methyl-D-aspartate-induced neurotoxicity by reducing calcium influx and acting on the cellular anti-oxidative defense system [15]. In this study, we examined the neuroprotective effect of DA against transient cerebral ischemic damage. This is the first study confirming the neuroprotective effect of DA in animal models of cerebral ischemia; however, we did not found any neuroprotective effects of 10 and 20 mg/kg DA administration. This finding is supported by a previous study that showed that 50 mg/kg DA did not protect against kainic acid-induced pyramidal cell death in the mouse hippocampal CA3 region, although DA attenuated kainic acid-induced lethal toxicity in a dose-dependent manner [28].

In the present study, we first examined neuroprotective effects of 10 and 20 mg/kg ASA-DA in the hippocampal CA1 region induced by transient global cerebral ischemia, and found that only pre-treatment of 20 mg/kg ASA-DA showed a significant neuroprotective effect compared with the other drugs. This finding is in line with a previous study, which reported that 100 mg/kg nitro-derivative of ASA, not 100 mg/kg ASA, treatment led to a significant reduction in infarct volume following focal cerebral ischemia in the spontaneously hypertensive rat [10]. Therefore, it is likely that ASA-DA treatment produced a synergistic neuroprotective effect against transient cerebral ischemic damage.

On the other hand, PET imaging is commonly used to examine brain glucose metabolism in patients [29]. We, in this study, compared glucose metabolism by PET-CT in the rat brain induced by transient focal cerebral ischemia between the vehicle- and ASA-DA-ischemia-group, and found that 20 mg/kg ASA-DA pre-treatment significantly maintained glucose metabolism in the brain induced by transient focal cerebral ischemia. Some researchers have already reported that neuroprotective effects of ASA or its derivatives were associated with an increase in brain ATP in some animal models of brain ischemia [9,30].

In addition, we found that 20 mg/kg ASA-DA pre-treatment significantly decreased the hyperactivity induced by transient global cerebral ischemia. Spontaneous motor activity is a good screen for behavioral change following transient cerebral ischemia in gerbils. It has been suggested that hyperactivity after ischemia-reperfusion in gerbils is predictive of neuronal loss in the hippocampal CA1 region, therefore, it is a useful method for evaluating neuroprotection against ischemic damage [31,32].

Cell death/damage triggers the activation of the immune system following ischemic insults, which is characterized by the activation of astrocytes and microglia together with the infiltration of peripheral immune cells [33,34]. In addition, it is well known that microglia and astrocytes are activated following ischemic stroke [35]. Recently, it was reported that a suppression of microglial activation and an attenuation of pro-inflammatory cytokines were closely related to the neuroprotective effect of ASA against transient focal cerebral ischemia [36]. In the present study, we found that transient cerebral ischemia-induced activations of astrocytes and microglia were markedly decreased by 20 mg/kg ASA-DA pre-treatment. Some researchers have reported that the down-regulation of astrocytes and microglia levels, as a marker for decreasing cerebral ischemic inflammation, offered a neuroprotective mechanism [37]. Therefore, our present finding suggests that ASA-DA pre-treatment reduces glial activation induced by transient cerebral ischemia, which can easily produce neuronal damage/death and neuroinflammatory events following ischemic damage.

Increased ROS levels are a major cause of tissue injury after cerebral ischemia [38]. SODs catalyze superoxide radicals into hydrogen peroxide, and that CAT and Gpx convert H2O2 to H2O under normal physiological condition [39,40]. Therefore, many researchers have focused on protective effects of antioxidants against cerebral ischemic damage [41,42]. In the present study, we found that 20 mg/kg ASA-DA pre-treatment well maintained or increased levels of antioxidants inculding SODs, CAT and Gpx in the CA1 pyramidal neurons induced by transient global cerebral ischemia. It was reporetd that increased SOD1 reduced oxidative DNA damage and subsequent DNA-fragmented cell death after ischemia in SOD1 transgenic mice [43] and that mutant SOD2 deficient mice showed a significant increase in superoxide anion production following cerebral ischemic injury [44]. In addition, Jung et al. (2009) reported that regulation of SOD2 activity showed a neuroprotective effect against cerebral ischemic damage in mice [45]. Furthermore, it was reported that Gpx played an important role in the protection of neural cells exposed to ischemic injury in the SOD1 transgenic mouse [39]. Therefore, our findings suggest that maintenance of antioxidants after 20 mg/kg ASA-DA pre-treatment may be associated with the neruoprotective effect of ASA-DA against ischemic damage.

In conclusion, pre-treatment with 20 mg/kg ASA-DA could protect neurons from transient focal and global cerebral ischemic damage, and the neuroprotective effect of ASA-DA must be much higher than that of ASA or DA alone under the same dosage. In addition, the neuroprotective effect of ASA-DA may be closely related to the attenuation of glial activation and maintenance of antioxidants. However, in this study, we could not demonstrate pharmacological properties of ASA-DA. Therefore, further studies need to investigate its pharmacological properties for further developing neuroprotective effects of ASA-DA in clinic.
